# Composition of Web Services Using Markov Decision Processes and Dynamic Programming

**DOI:** 10.1155/2015/545308

**Published:** 2015-03-22

**Authors:** Víctor Uc-Cetina, Francisco Moo-Mena, Rafael Hernandez-Ucan

**Affiliations:** Facultad de Matemáticas, Universidad Autónoma de Yucatán, Anillo Periférico Norte, Tablaje Cat. 13615, Apartado Postal 192, Colonia Chuburná Hidalgo Inn, 97119 Mérida, YUC, Mexico

## Abstract

We propose a Markov decision process model for solving the Web service composition (WSC) problem. Iterative policy evaluation, value iteration, and policy iteration algorithms are used to experimentally validate our approach, with artificial and real data. The experimental results show the reliability of the model and the methods employed, with policy iteration being the best one in terms of the minimum number of iterations needed to estimate an optimal policy, with the highest Quality of Service attributes. Our experimental work shows how the solution of a WSC problem involving a set of 100,000 individual Web services and where a valid composition requiring the selection of 1,000 services from the available set can be computed in the worst case in less than 200 seconds, using an Intel Core i5 computer with 6 GB RAM. Moreover, a real WSC problem involving only 7 individual Web services requires less than 0.08 seconds, using the same computational power. Finally, a comparison with two popular reinforcement learning algorithms, sarsa and *Q*-learning, shows that these algorithms require one or two orders of magnitude and more time than policy iteration, iterative policy evaluation, and value iteration to handle WSC problems of the same complexity.

## 1. Introduction

A Web service is a software system designed to support interoperable machine-to-machine interaction over a network, with an interface described in a machine-processable format called Web Services Description Language [1]. A Web service is typically modeled as a software component that implements a set of operations. The emergence of this type of software components has created unprecedented opportunities to establish more agile collaborations between organizations, and as a consequence, systems based on Web services are growing in importance for the development of distributed applications designed to be accessed via the Internet.

When a Web service is requested, all available Web services descriptions must be matched with the requested description, so that an appropriate service with the desired functionality can be found. However, since the number of available Web services is continuously growing year by year, finding the best match is not a trivial problem anymore, especially if we take into account that the matching criteria must consider not only the desired functionality, but also other attributes such as execution cost, security, performance, and so forth.

If individual Web services are not able to meet complex requirements, they can be combined to create composite services [2]. A composite Web service has one initial task and one ending task, and between the initial and the ending tasks there can be *k* = {0,1, 2,…, *K*} individual tasks connected in sequential order. To create a composite Web service it is necessary to discover and select the most suitable services. The complexity of WSC involves three main factors: (1) the large number of dynamic Web Services instances with similar functionality that may be available to a complex service; (2) the different possibilities of integrating service instance components into a complex service process; (3) various performance requirements (e.g., end-to-end delay, service cost, and reliability) of a complex service.

### 1.1. Related Work

Some approaches to solve the WSC problem have focused on different graph-based algorithms [3–8]. Some others have proposed to use optimization methods specially designed for solving constraint satisfaction problems, such as integer programming [9], linear programming [10], or methods for solving the knapsack problem [11]. Artificial intelligence methods such as planning algorithms [12–14], ant colony optimization [15], fuzzy sets [2], and binary search trees [16] have been used too.

The use of methods based on Markov decision processes (MDPs) for the composition problem is certainly not new. In [17], the problem of workflow composition is modeled as a MDP and a Bayesian learning algorithm is used to estimate the true probability models involved in the MDP. In [18], the WSC is solved using QoS attributes in a MDP framework with two versions of the value iteration algorithm: one backward and recursive and one forward version. In [19], the authors proposed the use of what they call value of changed information. Their approach uses MDPs focusing on changes of the state transition function, in order to anticipate values of the service parameters that do not change the WSC. In [20], a combination of MDPs and HTN (Hierarchical Task Network) planning is proposed.

Solutions based on reinforcement learning are also relevant. For instance, in [21], reinforcement learning and preference logic were employed together to solve the WSC problem, obtaining some kind of qualitative solution. Authors argue that computing a qualitative solution has many advantages over a quantitative one. Other methods using* Q*-learning are given in [22–24]. It is important to remember that reinforcement learning methods [25] belong to a family of algorithms highly related to the MDPs. The main difference with these methods is that the state transition function is assumed to be unknown and therefore the agents need to explore their state and action spaces by executing different actions in different states and observe the numerical rewards obtained after each state transition.

### 1.2. Contributions of This Paper

The goal of automatic WSC is to determine a sequence of Web services that can be combined to satisfy a set of predefined QoS constraints. For problems where we need to find the sequence of actions maximizing an overall performance function, the MDPs are one of the most robust mathematical tools that we can use. Therefore, in this paper we propose an MDP model to solve the WSC problem. To show the reliability of our model, we conducted experiments with three of the most studied algorithms: policy iteration, iterative policy evaluation, and value iteration. Although all three algorithms provided good solutions, the policy iteration algorithm required the minimum number of iterations to converge to the optimal solutions. We also compared these three algorithms against sarsa and* Q*-learning, showing that the latter methods require one or two orders of magnitude and more time to solve composition problems of the same complexity.

This paper is structured as follows.  Section 2 provides the basics of the MDPs framework and introduces the three algorithms that we tested.  Section 3 introduces our MDP model for solving the WSC problem.  Section 4 describes the experimental setup and presents the most relevant results.  Section 5 presents comparative experiments with sarsa and* Q*-learning algorithms. Finally, Section 6 concludes this paper by discussing the main findings and providing some advice for future research.

## 2. Markov Decision Processes

The WSC problem can be abstracted as the problem of selecting a sequence of actions, in such a way that we maximize an overall evaluation function. Such kind of sequential decision problems can be defined and solved in an MDP framework. An MDP is a tuple (*S*, *A*, *P*, *γ*, *R*), where *S* is a set of states, *A* is a set of actions, *P*(*s*_*t*+1_∣*s*_*t*_, *a*_*t*_) are the state transition probabilities for all states *s*_*t*_, *s*_*t*+1_ ∈ *S* and actions *a* ∈ *A*, *γ* ∈ [0,1) is a discount factor, and *R* : *S* × *A* → ℜ is the reward function.

The MDP dynamics is the following. An agent in state *s*_*t*_ ∈ *S* performs an action *a*_*t*_ selected from the set of actions *A*. As a result of performing action *a*_*t*_, the agent receives a reward with expected value *R*(*s*_*t*_, *a*_*t*_) and the current state of the MDP transitions to some successor state *s*_*t*+1_, according to the transition probability *P*(*s*_*t*+1_∣*s*_*t*_, *a*_*t*_). Once in state *s*_*t*+1_ the agent chooses and executes an action *a*_*t*+1_, receiving reward *R*(*s*_*t*+1_, *a*_*t*+1_) and moving to state *s*_*t*+2_. The agent keeps choosing and executing actions, creating a path of visited states *s*_*t*_, *s*_*t*+1_, *s*_*t*+2_,….

As the agent goes through states, *s*_0_, *s*_1_, *s*_2_,…, it obtains the following rewards:

(1) $$\begin{array}{c} R\left(s_{0},a_{0}\right)+\gamma R\left(s_{1},a_{1}\right)+\gamma^{2}R\left(s_{2},a_{2}\right)+\cdots . \end{array}$$

The reward at timestep *t* is discounted by a factor of *γ* ^*t*^. By doing so, the agent gives more importance to those rewards obtained sooner. In an MDP we try to maximize the sum of expected rewards obtained by the agent:

(2) $$\begin{array}{c} E\left[R\left(s_{0},a_{0}\right)+\gamma R\left(s_{1},a_{1}\right)+\gamma^{2}R\left(s_{2},a_{2}\right)+\cdots \right]. \end{array}$$

A policy is defined as a function *π* : *S* → *A* mapping from the states to the actions. A value function for a policy *π* is the expected sum of discounted rewards, obtained by performing always the actions provided by *π*:

(3) Vπs=Eγ2Rs0,πs0+γRs1,πs1   + γ2Rs2,πs2+⋯ ∣ s0=s,π.

*V* ^*π*^ is the expected sum of discounted rewards that the agent would receive if it starts in state *s* and takes actions given by *π*. Given a fixed policy *π*, its value function *V* ^*π*^ satisfies the Bellman equation:

(4) $$\begin{array}{c} V^{\pi }\left(s\right)=R\left(s,\pi \left(s\right)\right)+\gamma \underset{s^{\prime }\in S}{\sum }P\left(s^{\prime }\;|\;s,\pi \left(s\right)\right)V^{\pi }\left(s^{\prime }\right). \end{array}$$

The optimal value function is defined as

(5) $$\begin{array}{c} V^{*}\left(s\right)=\underset{\pi }{\max}\;V^{\pi }\left(s\right). \end{array}$$

This function gives the best possible expected sum of discounted rewards that can be obtained using any policy *π*. The Bellman equation for the optimal value function is

(6) $$\begin{array}{c} V^{*}\left(s\right)=\underset{a\in A}{\max}\left[R\left(s,a\right)+\gamma \underset{s^{\prime }\in S}{\sum }P\left(s^{\prime }\;|\;s,a\right)V^{*}\left(s^{\prime }\right)\right]. \end{array}$$

The optimal value function is such that we have

(7) $$\begin{array}{c} V^{*}\left(s\right)=V^{\pi^{*}}\left(s\right)\geq V^{\pi }\left(s\right). \end{array}$$

### 2.1. Dynamic Programming Algorithms for MDPs

When the state transition probabilities are known, dynamic programming can be used to solve (6). Next, we present three efficient algorithms for solving finite-state MDPs by means of dynamic programming. The first one is the iterative policy evaluation (given in Algorithm 1). The second one is the policy value iteration algorithm (given in Algorithm 2). This algorithm repeatedly computes the value function for the current policy and then updates the policy using the current value function. The third one, shown in Algorithm 3, called value function iteration, can be thought as an iterative update of the estimated value function using Bellman Equation (6).

The last two algorithms are known to converge usually faster than the first one. Moreover policy iteration and value iteration are standard algorithms for solving MDPs, and there is not currently universal agreement over which algorithm is better [26, 27].

## 3. Web Service Composition Model

In this section we define the MDP model used to represent and solve the Web service composition problem by means of dynamic programming algorithms.

We begin by describing the WSC problem in more details. Individual Web services can be categorized in classes by their functionality, input data, and output data. Given *C* different classes of individual Web services, the WSC problem consists in finding a sequence of length *C* of individual Web services 〈*w*_1_, *w*_2_,…, *w*_*C*_〉, such that *w*_*i*_ ∈ *W*_*i*_, for *i* = 1,2,…, *C*, where *W*_*i*_ is the set of all available Web services of class *i*. Thus, we are making the assumption that a valid composite Web service needs a Web service from each of the existing classes. We are also making the assumptions that all available Web services have been previously categorized into *C* classes and that the ordering of the classes *W*_1_≺*W*_2_≺⋯≺*W*_*C*_ has been predefined. *W*_*i*_≺*W*_*j*_ means that a Web service from set *W*_*i*_ must be executed before a Web service from set *W*_*j*_ to ensure the correct operation of the selected Web services. The correct operation depends basically on their functionality and input and output data. Therefore, the output of *w*_*i*_ must be fully compatible with the input of *w*_*j*_.

Now, we are ready to introduce our model. We define a Web service composition problem as an MDP (*S*, *A*, *P*, *γ*, *R*), where *S* is the set of states, *A* is the set of actions, *P* is the state transition probability function, *γ* is a discount factor such that *γ* ∈ [0,1), and *R* is the reward function. Elements *S*, *A*, *P*, and *R* are defined next.

### 3.1. States

*S* is the set of states. Given a WSC problem with *C* classes, *S* consists of all compositions of length at most *C*. Thus, for *C* = 1, *S* = {〈*w*_1_〉}, with *w*_1_ ∈ *W*_1_. A composition of length *l* = 1 is not really a composition; it is just a single Web service; however, we will relax the meaning of the word composition and will call it a composition of length *l* = 1. For *C* = 2, *S* = {〈*w*_1_〉, 〈*w*_1_, *w*_2_〉}, with *w*_1_ ∈ *W*_1_ and *w*_2_ ∈ *W*_2_. For *C* = 3, *S* = {〈*w*_1_〉, 〈*w*_1_, *w*_2_〉, 〈*w*_1_, *w*_2_, *w*_3_〉}, with *w*_1_ ∈ *W*_1_, *w*_2_ ∈ *W*_2_, and *w*_3_ ∈ *W*_3_. In general, for a WSC problem with *C* classes *S* = {〈*w*_1_〉, 〈*w*_1_, *w*_2_〉,…, 〈*w*_1_, *w*_2_,…, *w*_*C*_〉}.

### 3.2. Actions

*A* is the set of all actions. Given a state *s*, the set of actions available from *s* is denoted by *A*(*s*); thus *A* = {*A*(*s*)}_*s*∈*S*_. An action consists of selecting a Web service to be included in the current composition. If the current composition is of length *l* = *i*, all the possibilities of selecting a Web service of class *c* = *i* + 1 will constitute the set of current available actions.

Formally, we say that *A* = {*A*(*s*_*l*=0_), *A*(*s*_*l*=1_), *A*(*s*_*l*=2_),…, *A*(*s*_*l*=*C*−1_)}, where *A*(*s*_*l*=*i*_) is read as the set of actions available from a state representing a composition of length *l* = *i*. Note that *A*(*s*_*l*=0_) refers to set of actions available from a composition of length *l* = 0, which corresponds to the state where none of the Web services has been selected yet.

For example, if the current state represents the composition 〈*w*_1_, *w*_2_〉 which is of length *l* = 2, then *A*(*s*_*l*=2_) is given by all the possibilities of selecting a Web service of class *c* = 3. In other words, we are in a situation where we have already selected Web services from class *c* = 1 and class *c* = 2, and now we need to select a Web service from class *c* = 3.

### 3.3. Transition Probabilities

*P*(*s*′∣*s*, *a*) are the state transition probabilities for all states *s*, *s*′ ∈ *S* and actions *a* ∈ *A*, which are currently available from *s* and *s*′. Note that the probability of going from a state *s* = 〈*w*_1_〉 to the state *s*′ = 〈*w*_1_, *w*_2_〉 is 1. Meanwhile, the probability of going from the same state *s* = 〈*w*_1_〉 to a state *s*′ = 〈*w*_1_, *w*_2_, *w*_3_〉 is 0. In other words, we can only go from a composition state of length *l* = *i* to another composition state of length *l* = *i* + 1.

### 3.4. Reward Function

*R*(*s*′∣*s*, *a*) is the reward received when action *a* is executed and the environment makes a transition from *s* to *s*′. The reward function for our model is computed using three QoS attributes, as indicated in (8), which was originally proposed in [22]. The QoS employed are availability, throughput, and execution time:

(8) $$\begin{array}{c} R\left(s\right)=\frac{av^{s}-av^{\min}}{av^{\max}-av^{\min}}-\frac{{\text{time}}^{s}-{\text{time}}^{\min}}{\text{tim}{\text{e}}^{\max}-\text{tim}{\text{e}}^{\min}}+\frac{tr^{s}-tr^{\min}}{tr^{\max}-tr^{\min}}, \end{array}$$

where *av* ^*s*^, time^*s*^, *tr* ^*s*^ are the availability, average execution time, and throughput values for the last Web service added to the composition represented by state *s*. *av* ^min⁡^, time^min⁡^, *tr* ^min⁡^ and *av* ^max⁡^, time^max⁡^, and *tr* ^max⁡^ are the minimum and maximum values for all the Web services.

## 4. Experimental Evaluation

In this section we provide the results of our experimental comparison using two scenarios, one real and one artificial. The experiments that we present in this section were performed running policy iteration, iterative policy iteration, and value iteration algorithms, on an Intel Core i5 2.5 GHz processor, on Windows 8.1, 64 bits operating system, and 6 GB RAM.

### 4.1. Real Scenario

The WSC problem considered as our first experimental scenario consists of 2 classes of Web services. One class is about weather services that can be used to obtain the current temperature in a city. The other class is about Web services that can be used to convert temperatures from one metric unit to another, for example, from Fahrenheit to Celsius. In the class of weather services we considered 3 different Web services.

- National Oceanic and Atmospheric Administration (NOAA) Web service, available at http://graphical.weather.gov/xml/SOAP_server/ndfdXMLserver.php.
- GlobalWeather Web service, available at http://www.webservicex.net/globalweather.asmx.
- Weather channel Web service, available at http://api.wunderground.com/.

In the class of metric units conversion services we considered 4 different Web services.

(i) A simple calculator Web service such as the one available at http://www.dneonline.com/calculator.asmx. Since
  (9) $$\begin{array}{c} C=\frac{5*\left(F-32\right)}{9}, \end{array}$$
  we can use subtraction, multiplication, and division operations for the temperature conversion.
(ii) ConvertTemperature Web service, available at http://www.webservicex.net/ConvertTemperature.asmx.
(iii) TemperatureConversions Web service, available at http://webservices.daehosting.com/services/TemperatureConversions.wso.
(iv) TempConvert Web service, available at http://www.w3schools.com/webservices/tempconvert.asmx.

We obtained the QoS attribute values of all 7 Web services using a java program designed to get the attribute values with the following formulas:

(10) $$\begin{array}{c} \text{Availability}=\frac{C_{S}}{C_{T}}, \end{array}$$

where *C*_*S*_ is the number of successful calls to the Web service and *C*_*T*_ are the total calls,

(11) $$\begin{array}{c} \text{Execution}\;\text{time}=\frac{T}{C_{T}}, \end{array}$$

where *T* is the total execution time for all the *C*_*T*_ calls,

(12) $$\begin{array}{c} \text{Throughput}=\frac{C_{S}}{T}, \end{array}$$

with *C*_*T*_ = 50.

In order to obtain representative QoS values for the Web services, we made many measurements, several days in different moments of the day. We obtained the values for each parameter and measurement, and then we calculated the average values for the QoS parameters.

**Figure 1:**
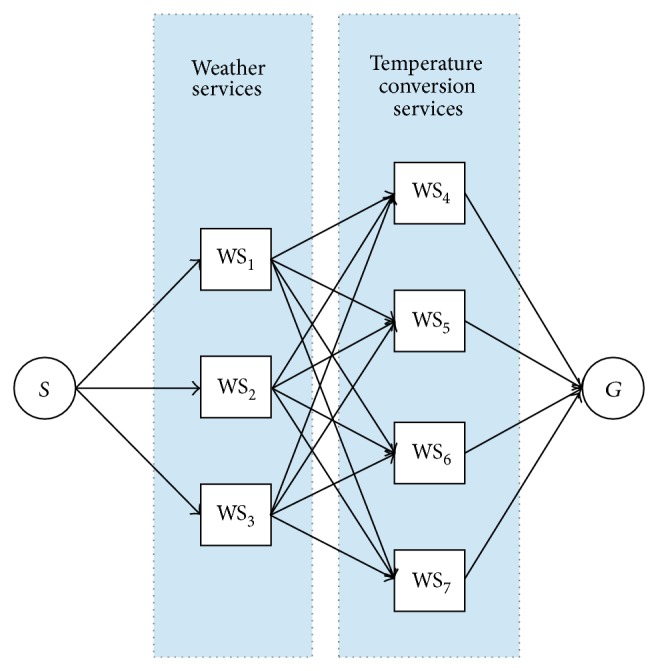
Graph for the real scenario with 2 classes of Web services. The first class contains 3 Web services and the second class contains 4 Web services. Each class is illustrated as a layer of nodes.

Once we gathered the information of the QoS attributes we used all 3 dynamic programming algorithms to learn the best composite Web service. With 7 Web services belonging to 2 different classes, there are 12 possible compositions. All these possibilities are represented with the graph illustrated in Figure 1.

The graph of the real scenario illustrates each class of Web services as a layer. In this graph, each node represents an individual Web service. Node *S* represents the state where none of the Web services has been selected yet. Node *G* represents the state where a full composition of Web service has been accomplished. A path from *S* to *G* implies that a valid composite Web service has been generated.

**Figure 2:**
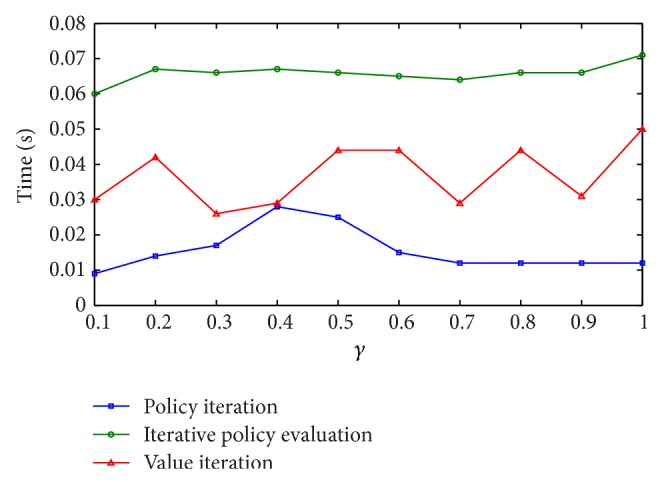
Learning times for the real scenario.

Results with the real Web services scenario are plotted in Figure 2. All 3 algorithms found the solution for the Web service composition very quickly, in less than 0.07 seconds, with policy iteration being the winner.

### 4.2. Artificial Scenario

**Figure 3:**
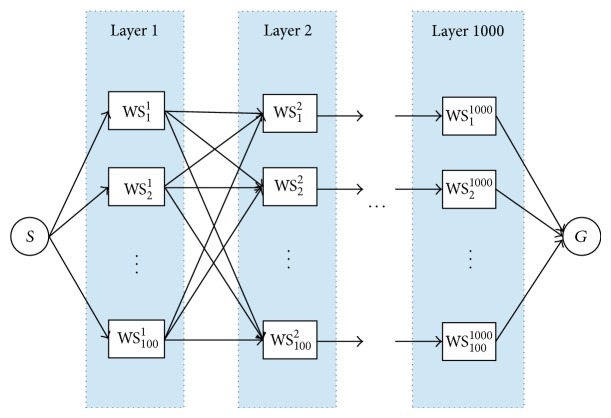
Graph for an artificially generated Web composition problem with a maximum of 1,000 selected nodes. Each node is selected out of 100 possible individual Web services belonging to the same class (layer).

As our second scenario to test all 3 dynamic programming algorithms, we simulated data for three QoS attributes: availability, execution time, and throughput. We created a maximum of 100,000 individual Web services, classified into 100 hypothetical classes of Web services. We assumed that every Web service in a class *i* can access all the Web services in class *i* + 1. Each of these classes is represented as a layer in Figure 3. Each layer contains 100 nodes or individual Web services.

As in the first scenario, node *S* is the initial state of the graph and represents a state where none of the Web services has been selected yet. Node *G* is reached when a valid composition has been accomplished. Nodes between *S* and *G* represent the available Web services. A route from *S* to *G* gives a possible composite Web service.

**Figure 4:**
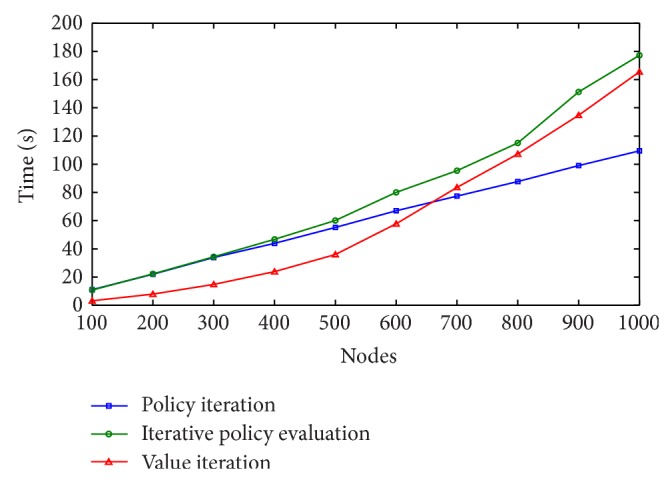
Learning times with *γ* = 0.7.

**Figure 5:**
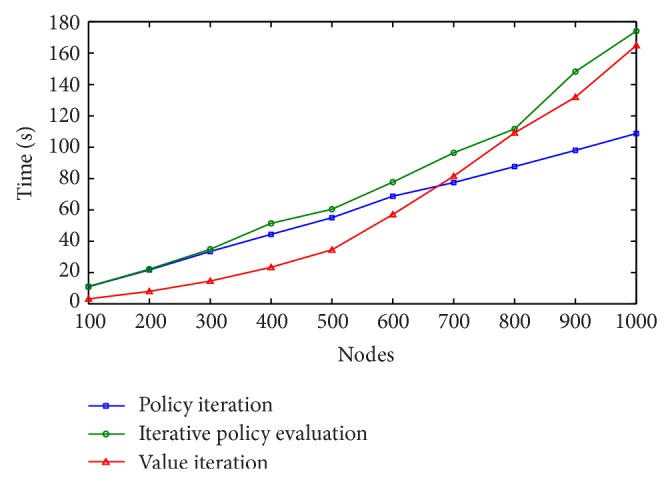
Learning times with *γ* = 0.8.

**Figure 6:**
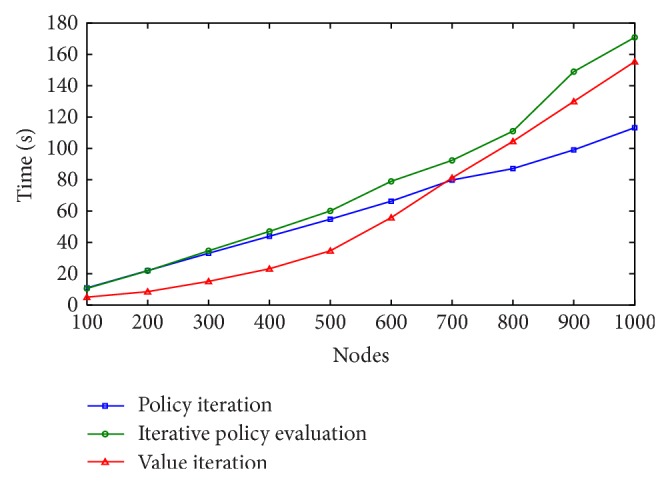
Learning times with *γ* = 0.9.

Results of this second set of experiments are shown in Figures 4, 5, and 6, for *γ* = 0.7, *γ* = 0.8, and *γ* = 0.9, respectively.

Each layer in the graph represents 100 Web services belonging to the same class. Therefore, when the number of nodes to be selected for a valid Web service composition is 1,000, we are really solving a problem with 100 × 1,000 = 100,000 Web services. We can see from the learning curves that the time needed to solve the MDP problem increases as the number of nodes is increased. Again, all 3 algorithms found the optimal solution, but policy iteration found it in less time. The best performances of the algorithms were obtained for *γ* = 0.8 and *γ* = 0.9, requiring less than 180 seconds to find the optimal composition using iterative policy evaluation and value iteration and less than 120 in the case of policy iteration.

## 5. Comparison with Sarsa and *Q*-Learning

In some related works [22–24], reinforcement learning algorithms were proposed to solve the Web service composition problem. In this section we compare the learning times required by sarsa and* Q*-learning against policy iteration, iterative policy evaluation, and value iteration.

### 5.1. Sarsa

Sarsa [25] is an on-policy temporal difference control algorithm which continually estimates the state-action value function *Q* ^*π*^ for the behavior policy *π* and at the same time changes *π* toward greediness with respect to *Q* ^*π*^.  Algorithm 4 presents the sarsa algorithm as taken from [25].

If the policy is such that each action is executed infinitely often in every state, every state is visited infinitely often, and it is greedy with respect to the current action-value function in the limit, then by decaying *α*, the algorithm converges to *Q* ^*^ [28].

### 5.2. Q-Learning

*Q*-learning [29] is an off-policy temporal difference control algorithm which directly approximates the optimal action-value function, independently of the policy being followed. It is one of the most popular algorithms in reinforcement learning.  Algorithm 5 reproduces the* Q*-learning algorithm as taken from [25].

If in the limit the action-values of all state-action pairs are updated infinitely often, with a decaying *α*, then the algorithm converges to *Q* ^*^ with probability 1 [26, 30].

### 5.3. Learning Time Analysis

**Figure 7:**
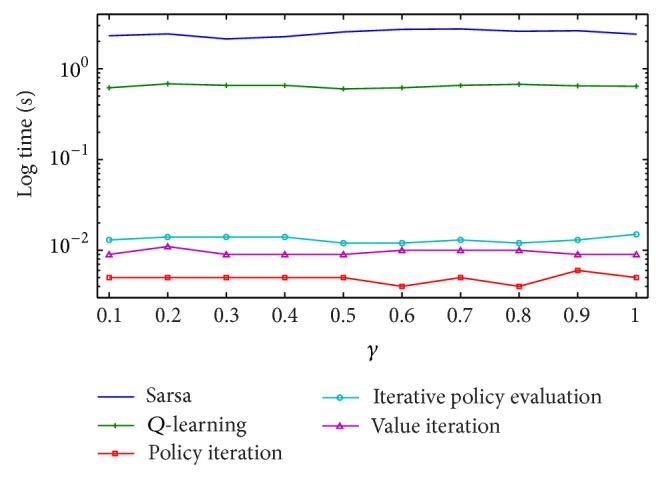
Learning times required for a real scenario of Web service composition, plotted in logarithmic scale. Reinforcement learning methods required two orders of magnitude and more time than dynamic programming methods.

We have implemented sarsa and* Q*-learning algorithms to solve the real scenario problem defined previously in the experimental section. A comparison graph illustrating the time required by sarsa,* Q*-learning, policy iteration, iterative policy evaluation, and value iteration is given using a logarithmic scale in Figure 7. From this graph we can clearly see that sarsa and* Q*-learning required two orders of magnitude and more time to find the optimal composition.

**Figure 8:**
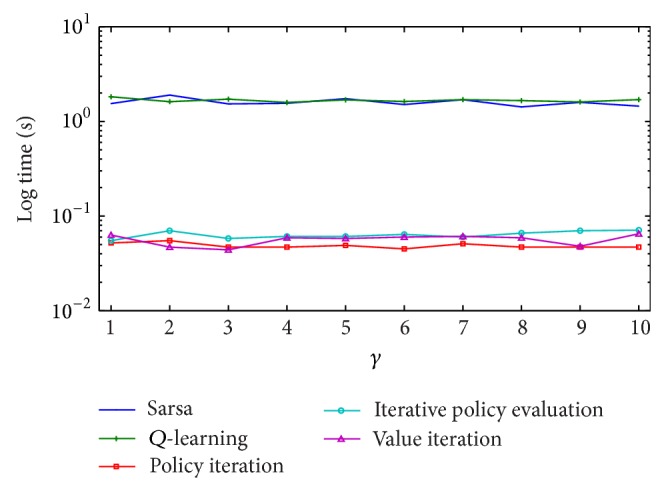
Learning times required for a simulated scenario with 3 layers of 20 Web services. Curves plotted in logarithmic scale show that reinforcement learning methods required ten times more time than dynamic programming algorithms to handle the same kind of problem.

Additionally, we ran experiments with a second artificially created scenario, with 3 layers of 20 Web services each. Once more, reinforcement learning methods required much more time than the dynamic programming algorithms. Logarithmic time curves given in Figure 8 show that sarsa and* Q*-learning required one order of magnitude and more time than dynamic programming algorithms. Furthermore, in some of the experiments, reinforcement learning algorithms failed to find the optimal solution, getting stuck in suboptimal compositions.

Dynamic programming methods converge faster than reinforcement learning methods simply because dynamic programming methods update every single state value at each iteration. Reinforcement learning methods only update the value of the states that happen to visit, giving its exploration policy, that is, epsilon greedy.

Furthermore, in terms of the deployment of an automatic Web service composition system, it is worth mentioning that the gathering of QoS information can be performed at specific time intervals by a dedicated module of such system. Once we have gathered this information, which is fundamental for the evaluation of the reward function, there is no need to explore the state space of Web services as reinforcement learning methods do. We can simply run a dynamic programming algorithm to estimate the value function of the Web services and then compute the optimal composition of Web services.

## 6. Conclusion

In this paper we have proposed an MDP model to address the Web service composition problem. We used three dynamic programming algorithms, namely, iterative policy evaluation, value iteration, and policy iteration, to show the reliability of our approach. Experiments were conducted with both artificially created data and a set of real data involving seven publicly available Web services.

Our experimental results show that policy iteration is the best one in terms of the minimum number of iterations needed to estimate an optimal policy. The optimal policy indicates the sequence of combined individual Web services making up a composite Web service with the highest evaluation of their QoS attributes.

Although some approaches using reinforcement learning have also been proposed, we argue that dynamic programming methods are better suited for the Web service composition problem than reinforcement learning methods. The reason is that reinforcement learning methods such as sarsa and* Q*-learning require a lot of exploration of the state space and consequently they need more iterations to make a good estimation of the optimal policy. To illustrate this, we compared sarsa and* Q*-learning against policy iteration, iterative policy evaluation, and value iteration. The result of this comparison is that sarsa and* Q*-learning required one or two orders of magnitude and more time than the dynamic programming methods to handle problems of the same complexity. Moreover, in some of the artificially created experiments, reinforcement learning algorithms got stuck in suboptimal Web services compositions.

None of the related works proposing the use of MDP-based methods to solve the Web service composition problem have provided a comparison study involving the five algorithms that we have analyzed in this work: iterative policy evaluation, value iteration, policy iteration, sarsa, and* Q*-learning. Moreover, we present experimental results using both a real scenario and a Web service composition scenario with artificially generated data. All other related works report experiments performed only with artificially created data.

Future research on this topic must address real Web services composition involving more nodes. Another interesting subject that deserves to be further investigated is the design of complex reward functions capable of handling an increasing number of QoS factors.

## Figures and Tables

**Algorithm 1:**
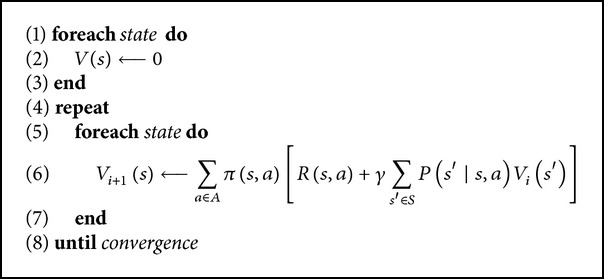
Iterative policy evaluation.

**Algorithm 2:**
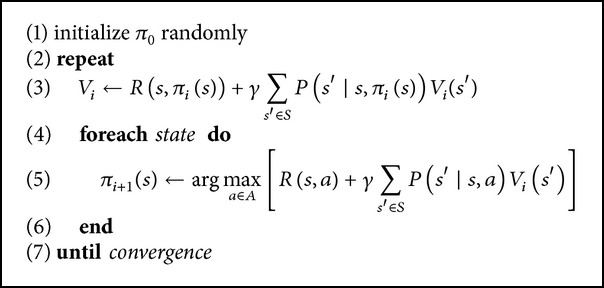
Policy iteration algorithm.

**Algorithm 3:**
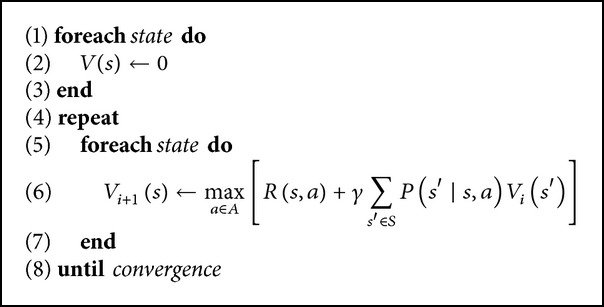
Value iteration algorithm.

**Algorithm 4:**
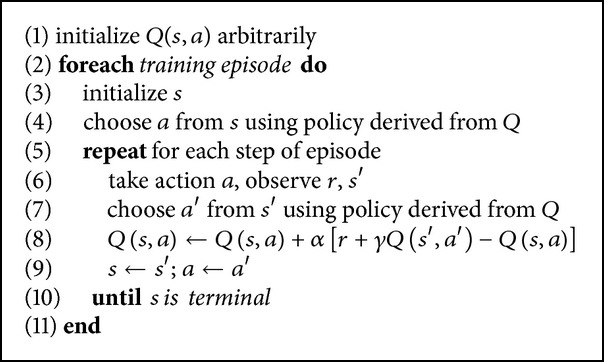
Sarsa algorithm.

**Algorithm 5:**
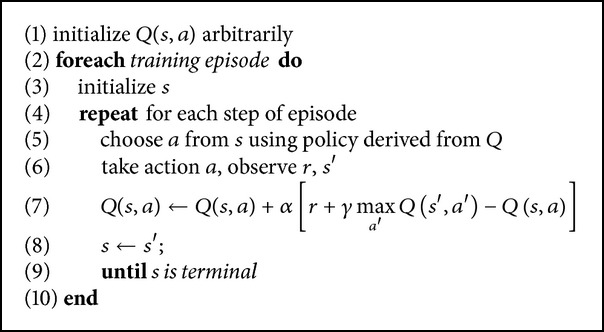
*Q*-learning algorithm.
